# Healthcare Provider N95 Respirator Contamination Worn Behind Face Shields With SARS-CoV-2 During Routine Clinical Care of Patients With COVID-19

**DOI:** 10.1093/ofid/ofae040

**Published:** 2024-02-02

**Authors:** Amanda M Graves, Bobby G Warren, Aaron Barrett, Sarah S Lewis, Becky Smith, David J Weber, Emily E Sickbert-Bennett, Deverick J Anderson

**Affiliations:** Division of Infectious Diseases, Duke Center for Antimicrobial Stewardship and Infection Prevention, Durham, North Carolina, USA; Division of Infectious Diseases, Duke University Medical Center, Durham, North Carolina, USA; Division of Infectious Diseases, Disinfection, Resistance and Transmission Epidemiology (DiRTE) lab, Durham, North Carolina, USA; Division of Infectious Diseases, Duke Center for Antimicrobial Stewardship and Infection Prevention, Durham, North Carolina, USA; Division of Infectious Diseases, Duke University Medical Center, Durham, North Carolina, USA; Division of Infectious Diseases, Disinfection, Resistance and Transmission Epidemiology (DiRTE) lab, Durham, North Carolina, USA; Division of Infectious Diseases, Duke Center for Antimicrobial Stewardship and Infection Prevention, Durham, North Carolina, USA; Division of Infectious Diseases, Duke University Medical Center, Durham, North Carolina, USA; Division of Infectious Diseases, Disinfection, Resistance and Transmission Epidemiology (DiRTE) lab, Durham, North Carolina, USA; Division of Infectious Diseases, Duke Center for Antimicrobial Stewardship and Infection Prevention, Durham, North Carolina, USA; Division of Infectious Diseases, Duke University Medical Center, Durham, North Carolina, USA; Division of Infectious Diseases, Duke Center for Antimicrobial Stewardship and Infection Prevention, Durham, North Carolina, USA; Division of Infectious Diseases, Duke University Medical Center, Durham, North Carolina, USA; Division of Infectious Diseases, University of North Carolina at Chapel Hill, Chapel Hill, North Carolina, USA; Division of Infectious Diseases, University of North Carolina at Chapel Hill, Chapel Hill, North Carolina, USA; Division of Infectious Diseases, Duke Center for Antimicrobial Stewardship and Infection Prevention, Durham, North Carolina, USA; Division of Infectious Diseases, Duke University Medical Center, Durham, North Carolina, USA; Division of Infectious Diseases, Disinfection, Resistance and Transmission Epidemiology (DiRTE) lab, Durham, North Carolina, USA

**Keywords:** healthcare epidemiology, hospital environment, mask contamination, SARS-CoV-2

## Abstract

N95 respirator contamination with severe acute respiratory syndrome coronavirus 2 (SARS-CoV-2) during clinical care of patients with coronavirus disease 2019 is poorly understood. We performed a prospective observational study on healthcare provider's (HCP’s) N95 respirators’ and face shields’ SARS-CoV-2 contamination during aerosol-generating procedures on SARS-CoV-2–positive patients housed in a COVID-19–specific unit. Medical masks worn on top of HCP's N95 respirators, and under face shields, during study aerosol-generating procedures were used as surrogates to detect contamination to avoid waste. Thirty-three HCPs were studied, and a total of 33 mask and 27 face shields were sampled. Masks were cut into 9 pieces and face shields were sampled twice, front and back, to determine locality of contamination; however, no positive samples were identified using standard polymerase chain reaction techniques with a CT value up to 40. All 9 mask piece samples were then pooled, as were face shield samples, using centrifugal concentration with polyethersulfone membranes. Once pooled and concentrated, overall, 9 (15%) samples were positive via real-time polymerase chain reaction: 5 from masks (15.2%) and 4 from face shields (14.8%).

## Teaser

We performed a prospective observational study to evaluate contamination of N95 respirators and face shields with SARS-CoV-2 when worn by healthcare providers giving care for SARS-CoV-2–positive patients housed in a COVID-19 unit. SARS-CoV-2 contamination was minimal.

The primary route of severe acute respiratory syndrome coronavirus 2 (SARS-CoV-2) transmission is respiratory droplets and aerosols [1–3]. During the coronavirus disease 2019 (COVID-19) pandemic, all people, from medical personnel to the general public, were advised to wear masks or face coverings (ie, face shields) to limit transmission of SARS-CoV-2. Within the healthcare setting, the Centers for Disease Control and Prevention currently recommends that healthcare providers (HCPs) wear an N95 respirator or powered air purifying respirator and additional personal protective equipment (PPE; ie, eye protection, gown, and gloves) when performing care on patients with known or suspected COVID-19 [4]. However, for most of the pandemic, HCPs were instructed to wear respirators and PPE for all types of care of patients with known or suspected COVID-19 that, at times, contributed to respirator shortages and implementation of novel strategies for reprocessing and reusing these respirators [5–7].

Contamination of N95 respirators with SARS-CoV-2 in HCPs treating patients with COVID-19 is not well described. Previous studies have demonstrated that respiratory viruses, including SARS-CoV-2, can be detected on respirators, masks, and PPE after use [8–12]. The Infectious Diseases Society of America recommends that HCP use face shields over N95 respirators when performing aerosol-generating procedures (AGP) to allow ongoing use of the N95 in a contingency/crisis scenario. As stated in the Infectious Diseases Society of America guidelines, this is a strong recommendation with low certainty of evidence [7]. To our knowledge, no study has evaluated mask contamination with the use of face shields during AGPs. The purpose of this study was to evaluate contamination of N95s worn by HCP when performing or present in the room during AGPs.

## METHODS

We performed a prospective observational study to evaluate contamination of N95 respirators and face shields with SARS-CoV-2 when worn by HCP providing care for SARS-CoV-2–positive patients housed in a COVID-19–specific unit at Duke University Hospital. HCPs were enrolled and participated in the study between September 2021 and March 2022. During the study period, N95 respirators were being reused because of the national shortage. Thus, surgical masks were worn on top of participating HCP's N95 respirators as surrogates for N95 respirator contamination to avoid waste, along with face shields, and evaluated after an AGP was performed. Each HCP was asked to don a surgical mask provided by the study team before entering the area where the AGP would take place, removed it after the procedure, during doffing, and given to the study team for microbiological analysis. HCPs were the same type of N95 respirators and face shields throughout the study. N95 respirators used were considered standard size, covering approximately 12 cm × 13 cm of the face. Disposable face shields used during the study measured 33 cm × 20 cm. Data collected included type of AGP, time of exposure, time spent in room, type of HCP, and proximity to patient during AGP.

### Study Population

This study was approved by Duke University institutional review board (Protocol 00108349). The HCPs asked to wear surgical masks over their N95s were providers entering a room where an AGP was taking place, which included nurses, physicians, respiratory therapists, certified nurse assistants, and other similar HCPs who may need to perform routine care in the study room. The HCPs provided role-specific routine care, such as assessing, administering medications, and maintaining oxygen supplementation while in patient rooms. HCP activities during the AGP were documented by study team members. Exposures varied on each AGP and when the HCP entered the room during the procedure. Study rooms were considered for inclusion when a room on a COVID-19–specific unit housed a positive patient on special airborne precautions and were chosen based on ordered AGPs. Frequently performed AGPs were targeted for inclusion (intubation, noninvasive ventilation, and high-flow oxygen). New study masks were provided to HCPs while donning PPE to enter a room with a COVID-19–positive patient on special airborne precautions; study masks were placed on top of existing N95 respirators.

### Sample and Clinical Data Collection

Study surgical masks were retrieved by study team members during doffing of PPE as the HCP exited the room removed gowns and double gloves, washing their hands, and regloved. Surgical masks were obtained by study team members and placed by HCP into biohazard bags. In addition, HCPs provided their face shields to be sampled during doffing, before standard disinfection. Face shields were swabbed in their entirety with nylon FLOQSwabs (Copan, Murrieta, California), using 1 swab for the front and another for the back, premoistened with viral transport media (VTM) (Redoxica, Little Rock, Arkansas) during doffing but before disinfection. Study team members collected data from routine clinical documentation, including patient COVID-19 symptoms, positive COVID-19 test date, AGP type, time of exposure/AGP, time the HCP spent in the AGP room, HCP type, HCP height, proximity and orientation of HCP to the patient, face shield dimensions, and possible contamination events to the medical masks.

### RNA Extractions and SARS-CoV-2 RT-PCR

**Figure 1.**
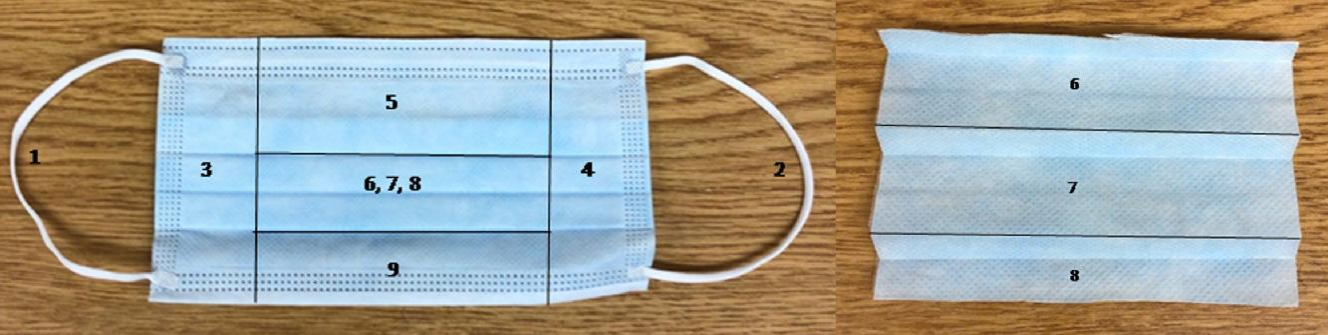
Nine position-based medical mask pieces.

Medical masks were cut into 9 position-based pieces, placed in VTM, vortexed, centrifuged, and separated into 2 separate samples; 1 sample was frozen for future use (Figure 1). Contamination was characterized by mask location (ear loop left, ear loop right, side left, side right, top, bottom, top middle, center, and bottom middle) and front and back of face shields. Face shield sample swabs were vortexed for 10 seconds to remove viral particles from the swab. RNA extractions were completed on the vortexed and centrifuged VTM using QIAamp Viral RNA Mini Kits (Qiagen, Hilden, Germany). Standardized and validated real-time polymerase chain reaction (RT-PCR) methods were completed on all samples using the US Centers for Disease Control and Prevention's 2019-nCoV Real-time RT-PCR assay protocol targeting the viral nucleocapsid (N) gene [13].

### Concentration of Samples

Despite using validated RT-PCR methodology for primary specimen assessment, all sample testing was initially negative. When our initial evaluations demonstrated no SARS-CoV-2 on any samples using conventional strategies, we wanted to determine if contamination was present but not being detected for methodologic reasons or because of very small concentrations of contamination being present. Thus, we reevaluated our microbiologic protocol to concentrate and identify the presence of virus. First, we validated a method for sample concentration and processing by adapting methods from several studies related to SARS-CoV-2 concentration [14–16]. Second, saved frozen samples were thawed. Third, all 9 samples from each medical mask were combined into a single sample, and both samples from each face shield were combined into a separate single sample. Then, these samples were centrifuged via concentrator centrifuge tubes, Vivaspin 20 1,000,000 MWCO (Sartorius, Bohemia, New York). Starting from approximately 18 mL of combined mask piece samples, the concentrator tubes were centrifuged at 3500 rpm for 10 minutes. The samples were concentrated 100 times to the adjusted volume of 180 µL. Finally, RNA extractions were completed on the concentrated samples from each consolidated mask using QIAamp Viral RNA Mini Kits (Qiagen). RNA extractions and RT-PCR were completed on all samples as previously intended.

### Statistical Methods/Analysis

This study was deemed exempt as nonhuman research by the Duke University Health System institutional review board. Study data were collected and managed using REDCap electronic data capture hosted at Duke University. The demographic characteristics of the study patients and HCP participants were summarized using descriptive statistics. The *Z* score proportionality test was used to compare proportions for samples with SARS-CoV-2 cycle threshold values. All statistical tests were 2-tailed and *P* < .05 was considered statistically significant. The data analysis was performed using SAS v9.1 software (Cary, North Carolina, USA).

## RESULTS

We enrolled 33 HCPs who provided care to 15 unique COVID-19–positive patients between September 2021 and March 2022. Patients’ median age was 67 (interquartile range [IQR], 60–76), and 10 (67%) were female. All patients were on high-flow nasal oxygen and actively symptomatic on the day of sampling: 8 (53%) fever, 11 (73%) cough, 11 (73%) shortness of breath, and 3 (20%) diarrhea. The median length of hospital stay was 8 days (IQR, 5–13) and the median length of stay in the study room was 7 days (IQR, 5–11). The median time from COVID-19–positive test and study evaluation was 5 days (IQR, 4–8) (Table 1).

**Table 1.** Characteristics of Healthcare Providers (HCP) and Patients

	N (%)
HCPs	N = 33
Registered nurse	12 (36)
Respiratory therapist	8 (24)
Physician	6 (18)
Speech pathology	2 (6)
Radiologic technologist	2 (6)
Phlebotomist	2 (6)
Certified nurse assistant	1 (3)
Height, cm (IQR)	165 (158–168)

Patients	N = 15
Age—median (IQR)	67 (60–76)
Female	10 (67)
Length of stay in room, days—median (IQR)	7 (5–11)
Symptoms	…
Shortness of breath	11 (73)
Cough	11 (73)
Fever	8 (53)
Diarrhea	3 (20)
Vaccinated^a^	6 (40)
Length between AGP and onset of symptoms, days—median (IQR)	11 (7–12)
Length between AGP and COVID-19+, days—median (IQR)	5 (3–6)

Abbreviations: AGP, aerosol-generating procedure; IQR, interquartile range.

^a^2 doses of mRNA vaccines, Pfizer or Moderna, or 1 dose of others, Johnson & Johnson.

A total of 33 masks and 27 face shields were evaluated. Before centrifugal concentration of medical mask pieces and the front and back of face shields, no positive samples were discovered. However, adding the concentration method resulted in 8 (13%) positive samples via RT-PCR from 7 HCP: 5 from masks (15.2%) and 3 from face shields (11.1%); 1 mask and face shield were from the same HCP. All 8 positive masks and face shields were obtained from HCPs with longer median time spent in the room than HCP with negative mask and face shield samples (median time in room = 18.5 minutes [IQR, 12.1–21.3] versus 13 [IQR, 8.0–28]; *P* = .68), although the difference was not statistically significant. In the rooms where positive samples from HCPs were obtained, AGPs were performed with a median length of 19 minutes (IQR, 13.5–28.75), compared with the median length of AGPs performed on negative mask samples (18.5 minutes; IQR, 8–40).

## DISCUSSION

In our study, healthcare providers’ face shields and masks were infrequently contaminated with COVID-19 virus even when performing AGP. When contamination was identified, it was only after high sample concentration and even then, at very low levels (ie, CT value >37) (Table 2). One reasonable conclusion is that face shields are largely preventing contamination. These findings support the Infectious Diseases Society of America recommendation and suggest that N95 respirators should not be considered contaminated if wearing a face shield, even after an AGP. This finding may be most relevant for contingency settings in which respirator conservation is necessary.

**Table 2.** SARS-CoV-2–positive Samples via RT-PCR After Sample Concentration Process

Sample Type	Ct N Gene
Face shield	38.01
Mask	37.50
Face shield	37.09
Mask	38.12
Mask^a^	39.10
Face shield^a^	37.86
Mask	37.68
Mask	37.21

Abbreviations: RT-PCR, real-time polymerase chain reaction; SARS-CoV-2, severe acute respiratory syndrome coronavirus 2.

^a^Positive samples from the same healthcare provider.

In general, our results are similar to previous studies. For example, Chughtai et al. found overall virus positivity (adenovirus, bocavirus, respiratory syncytial virus, and influenza) similar to this study with 10.1% of mask samples [8]. Similarly, Phan et al. found 12% of mask samples in their study were positive for respiratory viruses, such as influenza, rhinovirus, respiratory syncytial virus, and other coronaviruses [17]. However, neither of these studies evaluated contamination when a face shield was worn concomitantly. Our RT-PCR results generally support other studies completed during the COVID-19 pandemic. For example, Dargahi et al. did not find SARS-CoV-2 RNA on mask samples in an Iran hospital's COVID-19 patient ward worn by HCPs who were providing general care [9]. Few studies have examined respirator contamination during AGP. Shah et al. evaluated 51 masks and 38 face shields; 58 of the samples were obtained from PPE used in the care of COVID-19 patients, many of which underwent recent AGP. No samples in this study were positive for SARS-CoV-2; however, their results may have been impacted by HCPs disinfecting their face masks after donning and before the study team obtaining samples [10]. In contrast, our study evaluated face masks immediately after use and before disinfection. Additionally, it is important to note during the time period of the study, Delta and Omicron were the SARS-CoV-2 variants in our geographical region [18].

Our study has several limitations. First, our results may have been impacted by timing of evaluation because patients were several days into symptom onset at the time of AGP and evaluation. Although our results may not have reflected the highest risk periods for HCP exposure, they reflected the presumably high HCP exposure risk during AGP. Second, this study was completed in a COVID-19–specific unit, so results may not be generalizable to other healthcare environments. Similarly, precision of estimates is limited by small sample size, and selection bias could have been introduced because patients were not randomly selected. Last, we did not assess viral viability with cell culture for relatedness to index patients. However, CT values from our highly concentrated samples are suggestive that virus would not have been viable [3].

In conclusion, our results suggest that contamination of face shield and respirators of HCPs treating patients with COVID-19 undergoing AGP was minimal. This finding suggests that masks can be worn subsequently after AGP particularly in contingency scenarios.

## Notes

***Financial support*.** This work was supported by a grant from the Duke-UNC Prevention Epicenter Program for Prevention of Healthcare-Associated Infections—Coronavirus Preparedness and Response Supplemental Appropriations Act [grant no. U54CK00483].

## References

[1] Kampf G, Todt D, Pfaender S, Steinmann E. Persistence of coronaviruses on inanimate surfaces and their inactivation with biocidal agents. J Hosp Infect 2020; 104:246–51. 32035997 10.1016/j.jhin.2020.01.022 PMC7132493

[2] Colaneri M, Seminari E, Novati S, et al Severe acute respiratory syndrome coronavirus 2 RNA contamination of inanimate surfaces and virus viability in a health care emergency unit. Clin Microbiol Infect 2020; 26:1094.e1–.e5. 10.1016/j.cmi.2020.05.009 PMC7243766 32450255

[3] Warren BG, Nelson A, Barrett A, et al Severe acute respiratory syndrome coronavirus 2 environmental contamination in hospital rooms is uncommon using viral culture techniques. Clin Infect Dis 2022; 75:e307–9. 35023553 10.1093/cid/ciac023 PMC8807208

[4] 4. Centers for Disease Control and Prevention . 2022. Interim infection prevention and control recommendations for healthcare personnel during coronavirus disease 2019 (COVID-19) pandemic. Available at: https://www.cdc.gov/coronavirus/2019-ncov/hcp/guidance-risk-assesment-hcp.html#:∼:text=HCP%20should%20follow%20all%20recommended,SARS%2DCoV%2D2%20infection. Updated 23 September 2022. Accessed on 21 June 2022.

[5] Advani SD, Cromer A, Wood B, et al The impact of coronavirus disease 2019 (COVID-19) response on hospital infection prevention programs and practices in the southeastern United States. Infect Control Hosp Epidemiol 2023; 44:338–41. 34725004 10.1017/ice.2021.460 PMC8632447

[6] Schwartz A, Stiegel M, Greeson N, et al Decontamination and reuse of N95 respirators with hydrogen peroxide vapor to address worldwide personal protective equipment shortages during the SARS-CoV-2 (COVID-19) pandemic. Appl Biosaf 2020; 25:67–70. 36035079 10.1177/1535676020919932 PMC9387741

[7] Lynch JB, Davitkov P, Anderson DJ, et al Infectious Diseases Society of America Guidelines on Infection Prevention in Patients with Suspected or Known COVID-19. Infectious Diseases Society of America 2021; Version 2.0.0. Available at Available at: https://www.idsociety.org/practice-guideline/covid-19-guideline-infection-prevention/. Accessed 2 June 2023. 10.1093/cid/ciab953 PMC8767890 34791102

[8] Chughtai AA, Stelzer-Braid S, Rawlinson W, et al Contamination by respiratory viruses on outer surface of medical masks used by hospital healthcare workers. BMC Infect Dis 2019; 19:491. 31159777 10.1186/s12879-019-4109-x PMC6547584

[9] Dargahi A, Jeddi F, Ghobadi H, et al Evaluation of masks’ internal and external surfaces used by health care workers and patients in coronavirus-2 (SARS-CoV-2) wards. Environ Res 2021; 196:110948. 33684411 10.1016/j.envres.2021.110948 PMC7935683

[10] Shah A, Zhuang E, German J, et al Surface contamination of reusable respirators and face shields during care of critically ill COVID-19 patients. Workplace Health Saf 2023; 71:137–43. 36476243 10.1177/21650799221135583 PMC9742730

[11] Collins AP, Service BC, Gupta S, et al N95 respirator and surgical mask effectiveness against respiratory viral illnesses in the healthcare setting: a systematic review and meta-analysis. J Am Coll Emerg Physicians Open 2021; 2:e12582. 34746923 10.1002/emp2.12582 PMC8552225

[12] Kasloff SB, Leung A, Strong JE, Funk D, Cutts T. Stability of SARS-CoV-2 on critical personal protective equipment. Sci Rep 2021; 11:984 33441775 10.1038/s41598-020-80098-3 PMC7806900

[13] CDC . CDC 2019-novel coronavirus (2019-nCoV) real time RT-PCR diagnostic panel. Available at: https://www.fda.gov/media/134922/download? fbclid=IwAR1muBA9eS11if-uu70kaytxJghGzhydOlPTi4W7CZotrhquCKaT0VP3UFo. Updated 7 March 2023. Accessed on 21 June 2022.

[14] Jafferali MH, Khatami K, Atasoy M, Birgersson M, Williams C, Cetecioglu Z. Benchmarking virus concentration methods for quantification of SARS-CoV-2 in raw wastewater. Sci Total Environ 2021; 755:142939. 33121776 10.1016/j.scitotenv.2020.142939 PMC7553858

[15] Trottier J, Darques R, Ait Mouheb N, et al Post-lockdown detection of SARS-CoV-2 RNA in the wastewater of Montpellier, France. One Health 2020; 10:100157. 32835069 10.1016/j.onehlt.2020.100157 PMC7415170

[16] Dumke R, de La Cruz Barron M, Oertel R, et al Evaluation of two methods to concentrate SARS-CoV-2 from untreated wastewater. Pathogens 2021; 10:195. 33673032 10.3390/pathogens10020195 PMC7917696

[17] Phan LT, Sweeney D, Maita D, Moritz DC, Bleasdale SC, Jones RM. Respiratory viruses on personal protective equipment and bodies of healthcare workers. Infect Control Hosp Epidemiol 2019; 40:1356–60. 31668149 10.1017/ice.2019.298

[18] Centers for Disease Control and Prevention. 2023. CDC Museum COVID-19 Timeline. Available at: https://www.cdc.gov/museum/timeline/covid19.html. Updated on 15 March 2023. Accessed on 15 December 2023.

